# Supplementary material for: Are carbon emissions trading and green financial instruments synergistic? -Comprehensive quantitative research based on content analysis
Source: PLoS One. 2024 Mar 7;19(3):e0298601. doi: 10.1371/journal.pone.0298601 (PMC10919601; doi:10.1371/journal.pone.0298601)
Supplement: S1 Data — (ZIP) [file pone.0298601.s001.zip › Data/地级市Z_F工作报告.zip!/║┌┴·╜¡/╥┴┤║╩╨/└┤╫╘╣1⁄2╓┌║┼í╛┬φ┐╦╩2╛▌═°í┐_2007.docx]

各位代表：

现在，我代表市政府，向大会作工作报告，请予审议，并请市政协各位委员和其他列席人员提出意见。

一、过去四年工作的回顾

市十一届人民代表大会第一次会议以来，在省委、省政府和市委的正确领导下，市政府团结带领全市人民，全面落实科学发展观，同心同德，奋发进取，较好地完成了市十一届人大各次会议确定的主要任务，实现了经济持续快速发展，社会事业全面进步。四年来，全市生产总值年均增长10.3%，规模以上工业增加值年均增长13.2%，地方财政收入年均增长11.8%，固定资产投资年均增长28.1%。

生态建设取得显著成果。深入实施“生态立市”战略，创造性地推进“天保工程”，把“严管林”作为生态建设的首要任务，提出并严肃执行“三个决不”原则和“四严四不”方针，有效地遏制了法人超限额采伐，严厉打击了盗伐、毁林等违法行为，清理整顿了木材加工厂点。全面停止天然红松林木采伐，保持了“红松故乡”的美誉。这些重要举措不仅保护了森林资源、促进了森工企业管理水平的明显提高，而且对调整产业结构、提高木材经济效益、增加财政收入、获得国家政策支持都起到了基础性作用。森林培育步伐加快，封山育林51.8万公顷，更新造林4.75万公顷，平均成活率达92.3%，营造民有林1.3万公顷，森林抚育25.1万公顷，建成各级各类自然保护区57.5万公顷、省级以上森林公园25个，野生动植物得到保护，森林覆被率由2002年的78.4%提高到2006年的80.6%，历史性地实现了森林资源年均净增长500余万立方米的良性消长循环，为解决我市资源性矛盾创造了良好的开端。森林防火工作成效显著，连续三年未发生重特大森林火灾。土地整理复垦和地质环境治理工作得到加强，获得国家和省资金1.4亿元，实施了8个土地整理复垦项目和11个地质环境保护项目。

各项改革稳步推进。经过多年的运筹，特别是2003年以来的积极争取，国家林业局于2004年4月正式批准我市为国有林区林业产权制度改革试点单位，2006年1月4日，国务院第119次常务会议原则通过在伊春进行国有林权制度改革试点的意见，2006年4月29日，一开先河的国有林权制度改革试点在我市正式启动，拉开了中国国有林权制度改革的序幕，5个试点局8万公顷流转任务于2006年底顺利完成，为解决我市体制性矛盾迈出了突破性一步。国有林权制度改革被评为2006中国十大改革案例奖。国有中小企业产权制度改革成效显著，森工企业附属单位实施了主辅分离、辅业改制。西钢集团改制顺利实施。西林铅锌矿政策性破产全面终结。晨明水泥厂政策性破产项目得到国务院批复。完成了政府机关机构改革工作。事业单位改革试点正在进行。行政审批制度改革不断深化。教育体制、财政管理、农村税费、粮食流通体制等各项改革都取得了成效。

经济转型工作取得新突破。2005年5月17日，我市被确定为全国唯一的林业资源型城市经济转型试点单位，为实现林业经济向林区经济转变提供了发展机遇。产业结构进一步优化，在重点培育和发展木材精深加工、森林生态旅游、绿色食品、生态畜牧、北药开发五大优势特色产业基础上，又加快发展了绿色能源、冶金建材化工、矿产开发等产业，接续替代产业呈现出竞相发展态势。带岭大箐山风力发电、西钢长流程改造、浩化10万吨甲醇、汤旺河5万立方米刨花板等大项目相继投产，加快了“工业兴市”的进程。接续替代产业增加值占全市GDP比重32.7%，全部工业增加值对地区生产总值增长的贡献率达到39.9%。所有制结构调整加快，非国有经济发展迅速，增加值由2002年的56.2亿元增加到2006年的94.9亿元，年均增长12.9%。从事自营经济的职工家庭14.8万户，年收入2万元以上的达6万户。

新农村建设初见成效。“一免两补”等各项支农惠农政策得到落实。农业经济结构进一步优化，粮食产量由2002年的36.3万吨增加到2006年的52.7万吨，有机、绿色、无公害食品种植面积达到150万亩，农业增加值年均增长10.1%。农业综合机械化程度达到81.4%，建立农业信息互联网节点2542个。15.1万人（次）农村义务教育阶段贫困学生享受到了“两免一补”政策。新型农村合作医疗制度试点进展顺利，农民参合率达78.5%。投入3.52亿元用于农村水利、公路建设和农业机械化更新，新建饮水工程166处，解决了9.1万人的饮水难问题。启动了省、市两级新农村建设试点54个，省、市财政和帮建单位对两级试点共投入资金2.25亿元，农村和林场所面貌发生了很大变化。

对外开放迈出新步伐。成功举办了四届森林生态旅游节、两届五花山观赏节等节事活动。组织企业参加了“哈洽会”、“香港活动周”、“韩国活动周”等一系列经贸洽谈会，有针对性地在广东、福建、浙江、北京、上海等地建立了招商引资基地，提高了招商引资的实效性。四年累计国内引资62.95亿元、实际利用外资2278万美元、实现外贸进出口总额2.6亿美元，年均分别增长47.5%、56.6%、26.9%。对俄经贸合作取得实质性突破，嘉荫口岸累计过货10万吨，乌马河等区（局）与国内外集团公司合作赴俄开发森林资源，采伐木材60.1万立方米，对俄劳务输出人数4829人。

生态园林城市建设进程加快。围绕“世界知名、中国一流、中等规模、生态园林”的城市建设目标，采取团组式建设、“曲径通幽”式连接措施，加快了“四区连片”步伐，扩大了中心城区规模，实现了新区加快发展，老区持续繁荣。基础设施建设不断加强，城市建设完成投资15.3亿元，新建经济适用住房109万平方米、市政道路185万平方米，完成漏雨楼房平改坡32万平方米，铺设供水管线228公里，新增集中供热面积318万平方米、公共绿地面积77公顷。建成了木雕园、兴安桥等一大批精品园林工程、景区景点及休闲场所，初步形成了山、水、林、城交相辉映的生态园林城市格局。兴安湖水库、垃圾处理厂和污水处理厂已开工建设，伊春林都机场被批准立项。累计投资21.58亿元，完成伊嘉公路及通乡通村公路1559.2公里，结束了伊春主干道砂石路的历史，“一纵三横一环”的公路主干线骨架网初步形成。建设运输场站48个。区（局）址城镇改造、生态林业局和生态林场所建设成效显著，加快了伊春整体向生态园林城市迈进的步伐。我市相继被评为国家级园林城市、全国绿化先进城市、中国人居环境范例奖、中国最佳生态旅游城市、中国最佳避暑胜地、中国旅游竞争力百强城市，被联合国国际交流合作与协调委员会评为“城市森林生态保护和可持续发展范例”，并授予“绿色伊春”荣誉称号。

人民生活水平不断提高。城镇居民人均可支配收入、农民人均纯收入年均分别增长7%、10.9%。城乡居民储蓄存款余额186.9亿元。社会消费品零售总额年均增长7.6%。累计实现就业再就业17万人,开发公益性岗位8000多个。一次性安置森工企业富余人员10.6万人，地方国有企业下岗职工基本生活保障向失业保险并轨2.6万人。企业职工基本养老保险实现了省级统筹，补发拖欠养老金1.2亿元，企业离退休人员养老金人均增加220元。符合条件的城镇低保对象基本实现了应保尽保，10391名农村困难群众纳入低保范围。12115户特困职工家庭通过扶贫解困工程实现脱贫，脱贫率达到98%。为企办教师增加了课时补贴，先后两次增调森工在岗职工工资，月人均工资增加到现在的414元。困难群体的保障水平和低收入者的收入水平有了新提高。

各项社会事业全面发展。科技创新能力进一步提高，取得科技成果116项，组织实施科技项目523项，新增产值8.2亿元。信息产业加快发展，自主研发的“数字林业”、“森林防火指挥系统”、“森林资源管护系统”得到推广应用。教育事业得到空前重视，投入不断增加，调整优化了中小学校布局，改造和新建校舍22.8万平方米，素质教育全面推进，基础教育质量提高，职业教育和高等教育不断加强。卫生事业稳步发展，建立了疾病预防控制、医疗救治等公共卫生体系，预防和应对突发公共卫生事件的能力进一步增强。低生育水平持续稳定，人口素质进一步提高。文化事业繁荣发展，创作出话剧《青山常在》等一大批优秀文艺精品，“大森林之声”系列文化活动丰富多彩。广播、电视、报刊充分发挥了新闻媒体作用。体育事业蓬勃发展，竞技体育成绩显著，在国际、国内大型比赛中获奖牌573枚。城市环境得到有效改善，工业废水、烟尘排放达标率、固体废物综合处置利用率分别达到91%、91.3%、72%，空气质量达到国家二级以上标准354天。防汛抗旱、水资源节约保护等水务工作得到加强。经济普查工作顺利完成。群众性的精神文明创建活动形式多样。“四五”普法任务圆满完成，平安伊春建设取得成效，被评为全国社会治安综合治理优秀城市。民族、宗教、外侨、档案、市志、边防、人防、城防等各项工作都取得了新成绩。

2006年是实施“十一五”规划的第一年，全市上下开拓创新，真抓实干，经济社会发展速度进一步加快。全市生产总值实现130.3亿元，同比增长11.1%。工业实现增加值40.2亿元，同比增长16.5%。财政总收入6.2亿元，同比增长17%，其中地方财政收入3.4亿元，同比增长15.4%。国内引资25.1亿元、实际利用外资986万美元、实现外贸进出口总额9180万美元，分别完成计划的136.4%、109.6%、112.4%。社会消费品零售总额达到28.9亿元，同比增长9.9%。全社会固定资产投资完成38.4亿元，同比增长30.3%。城镇居民人均可支配收入7416元，农民人均纯收入4135元，同比增长6%、9.1%。实现就业再就业4.3万人，城镇登记失业率4.4%。实行木材全面竞价，阳光操作，大幅度提高了销售价格。移植大树进城10万株。完成公路建设861公里，汤嘉路、伊春区北出口路、嘉荫口岸路、乌带路交付使用。伊春电力总公司成功转型为国家电网企业。建筑面积4500平方米的行政服务中心竣工并投入使用。地方及林业政企合一区（局）机关事业单位职工调资工作全面启动。退休职工养老金和城市低保标准有了新的提高。

各位代表、同志们，过去的四年，是全市森林资源得到有效保护和发展的四年，是经济加快发展、社会各项事业全面进步的四年，是人民群众享受改革发展成果受益较多的四年。成绩来之不易，令人鼓舞。这是省委、省政府正确领导和亲切关怀的结果；是全市人民在市委的领导下与时俱进、艰苦奋斗的结果；是各级人大依法监督、关心支持，各级政协民主监督、参政议政的结果。在此，我代表市人民政府，向全市广大工人、农民、知识分子、干部、离退休老同志、个体私营业者、域外投资客商，向人大代表、政协委员和各民主党派、工商联、无党派爱国人士、社会团体，向驻伊部队、武警官兵、公安司法干警、中省直驻伊单位，向所有关心支持伊春建设和发展的同志们、朋友们致以崇高的敬意和衷心的感谢！

过去的四年，我市经济社会发展虽然取得了喜人的成就，但还存在不少矛盾和问题。主要是：破坏森林资源和超限额采伐现象仍有发生，解决资源性的矛盾仍需做出更大的努力；经济总量小，外向度不高，财政保障能力不强；工业化水平低，规模企业拉动作用不明显，经济增长方式粗放；传统产业比重大，科技含量低，接续替代产业支撑能力不强；就业压力较大，社会保障体系不够完善，社会事业发展相对滞后；林业职工群众收入较低，困难群众较多；一些地方和部门不廉洁现象不同程度存在，经济发展环境还待进一步改善。对这些问题我们必须高度重视，采取有效措施，努力加以解决。

各位代表、同志们，总结四年的实践，我们深切体会到：第一，推动伊春全面发展，必须抓住事关全局的主要矛盾。我们以对伊春的历史和未来负责的态度，在对伊春发展历程进行深刻反思和当前发展趋势正确判断的基础上，从主要矛盾和矛盾的主要方面进行突破。通过严管资源和停伐红松的刚性措施，挽救、培育和壮大了森林资源，不但为促进林区经济发展奠定了根基，而且还凸显了小兴安岭的生态地位，为保护生态环境创造了范例，引起了上级乃至联合国有关组织的高度重视和全社会的关注支持，使“红松故乡”、“绿色伊春”声名远播，为破解历史遗留问题和发展难题打下了基础。第二，推动伊春全面发展，必须牢牢抓住改革这个主题。我们通过不断深化对市情的认识、再认识，大胆从改革上找出路，抓住体制机制难点搞突破。敢为人先，勇担风险，率先在国有林区实施了林权制度改革试点，为国有林区现行森林资源管理体制改革探索新路，抓住了改革主题,使有限的林木、林地等林业生产要素激活起来，极大地解放和发展了林业生产力，通过改革，为解决养林与养人、发展经济与保护资源的历史性难题创造了新模式，为林区广大职工群众尽快致富开辟了新的途径。第三，推动伊春全面发展，必须紧紧抓住维护人民群众利益这个根本。我们始终把维护群众利益作为政府一切工作的出发点和落脚点，着力解决就业、社保、教育、住房、工资等民生问题，让人民群众在改革和发展中得到实惠。尤其是通过创新方式，采取引资、置换、争取、招商、BOT等办法，拓宽了城市建设融资渠道，明显改善了人居环境。心系群众、真情为民的实际行动，赢得了广大群众的信任和支持，调动了全市上下干事创业的积极性，形成了思想统一、目标一致、合力发展的良好局面。第四，推动伊春全面发展，必须善于争取政策解决经济社会发展中的难点问题。伊春为国家做出了重大历史贡献，也出现了一些自身难以解决的困难和问题。我们积极抓住机会，利用机会充分反映情况，对上争取，求得国家认可，通过国家的关怀来帮助伊春破解经济转型难题，从而为伊春赢得了更高层次和方方面面的关注，获得了前所未有的直接支持和政策惠及，有力地推动了经济结构战略性调整和社会事业的全面进步，为伊春今后的发展增添了后劲。第五，推动伊春全面发展，必须坚持用发展的办法解决发展中的问题。为了破解长期积淀的“四大矛盾”，我们始终立足林区实际，突出发展第一要务，确立了“生态立市”和“工业兴市”战略，积极发展优势特色产业，大力开展招商引资，强力推进大项目建设，做到在保护中发展，在发展中保护，使经济运行速度、效益和质量得到全面提升，实现了历史上的新突破。实践证明，发展是解决一切问题的关键所在。只要全市上下坚定加快发展的信心和决心不动摇，一切着眼发展，一切围绕发展，用发展的实绩来检验我们的工作，凝心聚力，真抓实干，全市经济社会就一定能够实现新的跨越。

二、今后五年的奋斗目标、主要任务和2007年工作安排

今后五年全市经济社会发展的总体要求是：以邓小平理论和“三个代表”重要思想为指导，全面落实科学发展观，深入实施市十次党代会确定的发展战略和“十一五”规划，加大森林资源保护培育力度，积极推进以国有林权制度改革为重点的各项改革，进一步扩大开放，增强自主创新能力，调整经济结构，转变增长方式，加速推进经济转型，强化社会管理，改进公共服务，着力解决民生问题，提高人民群众物质文化生活水平，加快构建林区和谐社会，努力实现经济社会又好又快发展，全面建设社会主义新林区。

根据上述要求，今后五年我市经济社会发展的主要预期目标是：

----森林资源得到进一步恢复。到2011年森林总蓄积量由 2.28亿立方米增加到2.6亿立方米，人工林累计保存面积50万公顷，森林覆被率达到80.8%。

----综合经济实力显著增强。到2011年全市生产总值达到 210亿元，年均增长10%，人均GDP达到1.68万元；地方财政收入达到5.4亿元，年均增长10%；固定资产投资年均增长19%以上，累计达到270亿元。

----经济结构更加优化。三次产业比重调整为22：32.8：45.2；木材精深加工、生态旅游、特色种养与加工、冶金建材及矿产开发、绿色能源五大接续替代产业增加值占GDP的比重达到50%以上，非国有经济增加值年均增长12%；单位生产总值能源消耗年均降低4%。

----城乡协调发展。随着政策的到位，建立无人区，实施城镇重组，到2011年中心城区面积达到200平方公里，人口达到50万，人均绿地面积达到61.67平方米；城市绿化覆盖率达到70%；新农村建设取得明显成效，城乡差距进一步缩小。

----人民生活水平大幅提高。城镇居民人均可支配收入年均增长8%，农民人均纯收入年均增长6%；城乡居民最低生活保障覆盖率达到97%；城镇从业人员基本养老、医疗、失业保险覆盖率达到100%，新型农村合作医疗参合率达到85%以上；城镇登记失业率控制在3.5%以内，新增就业岗位10万个；城镇人均住房面积达到20平方米，农民生活用房面积达到22平方米。

----社会事业和谐发展。教育事业协调均衡发展，高中阶段教育逐步普及，中高等职业教育稳步发展；科技创新能力进一步增强，科技贡献率提高到40%以上；公共卫生体系逐步完善，服务水平进一步提高；文化事业繁荣发展，生态文化建设取得明显成效；体育事业长足发展，竞技体育水平进一步提高；精神文明建设得到加强，社会更加安定有序和谐。

实现上述目标，保持经济社会又好又快发展，必须坚持以科学发展观为统领，做到“六个更加注重”：

一是更加注重生态建设。继续实施“生态立市”战略，加快建设小兴安岭生态功能区和生态经济区。争取扩大延伸“天保工程”，调减木材产量，培育扩大森林资源。保护好河流、湿地和野生动植物资源，维护生物的多样性。恢复和增强森林生态系统的整体功能，逐步建立具有小兴安岭特色的比较完备的生态体系。处理好经济建设与生态保护、资源利用之间的关系，大力发展生态经济、循环经济，探索发展林业碳汇项目，全面推进节约发展、清洁发展、安全发展，逐步建立具有小兴安岭特色的生态型经济体系，促进经济、社会、生态三大效益的协调统一。

二是更加注重走新型工业化道路。坚定不移地实施“工业兴市”战略，坚持以信息化带动工业化，以工业化促进信息化。突出抓好项目建设，做强做大优势特色工业，培育一批知名度高、竞争力强、对财政贡献大、出口创汇的骨干企业、名牌企业和名牌产品。

三是更加注重体制机制创新。继续深化各项改革，着力解决体制性矛盾。重点抓好国有林权制度改革，扩大试点范围。深化国有企业改革。优化主导产业资源配置，逐步实现集团化经营。帮助引导规模以上民营企业进行股份制改造，建立现代企业制度。推进政企分开、政事分开。深入搞好农村综合改革。实现体制比较先进、机制比较灵活的改革目标。

四是更加注重城乡统筹发展。以改善人居环境、争创国家级生态园林城市为目标，以提质扩容为重点，加大中心城区和小城镇建设力度，建设环境优美的森林城市，彰显山水个性，展示林都魅力。加快社会主义新农村建设，优化农业产业结构，发展现代农业，提高农业综合效益，增加农民收入，加快农村基础设施建设和公共事业发展，初步形成以城带乡、城乡互动、协调发展的格局。

五是更加注重对外开放。实施互利共赢的开放战略，改善投资环境，建立招商引资长效机制，重视企业在招商引资中的主体作用，采取灵活多样的形式，扩大招商引资成果，提升引资的质量。继续推进对俄经贸科技合作战略升级，向宽领域、高层次、纵深化发展。着力扩大外贸出口，优化出口产品结构，提高外经外贸水平，推动全面对外开放。

六是更加注重和谐社会建设。强化民主法制建设，保障人民在政治、经济、文化、社会等方面的权利和利益。尽最大努力解决就业、就医、就学难和收入低、保障低等与人民群众切身利益密切相关的问题，逐步建立养老保险、社会救助、社会福利、慈善事业相衔接覆盖城乡居民的社会保障体系，使民生状况有较大的改善。完善社会管理，保持社会安定有序。

2007年是新一届市政府团结带领全市人民站在新起点、抢抓新机遇、谋求新发展、实现新跨越的起始之年。做好2007年的工作，为今后五年的发展开好局、起好步具有重大意义，必须突出重点，狠抓落实。

2007年我市经济社会发展的主要预期目标是：全市生产总值同比增长10%。地方财政收入按可比口径增长10.2%。全社会固定资产投资增长25%以上。社会消费品零售总额增长11%。国内引资、实际利用外资、外贸进出口分别比上年实际完成增长20%。万元GDP综合能耗降低5%。城镇居民人均可支配收入增长8%，农民人均纯收入增长6%。城镇居民登记失业率控制在4%以内。

为实现上述目标，重点抓好以下8个方面的工作。

（一）积极培育保护森林资源，加快小兴安岭生态功能区建设

继续严管资源。深入实施“天保工程”，坚定不移地执行“严管林”方针，强化采伐限额管理，杜绝法人超采，严惩偷拉私运、毁林盗伐等涉林违法行为，严肃追究超采单位法人代表及相关责任人的行政或刑事责任。在全面停伐天然红松林木的基础上，与建设银行合作，年内发行“保护红松信用卡”，建立红松基金，引领全社会关注红松，开展支持保护和恢复红松行动。强化林政监督检查，实行“漏斗式”管理，整顿木材加工市场秩序。提高木材经销管理水平，全面推行木材联网竞价销售，促进木材增收。加强林地管理，从严控制征用占用林地，坚决制止农林交错地带蚕食林地行为，积极组织恢复废弃林地森林植被，防止林地逆转。严厉打击滥捕乱猎、滥采乱挖不法行为，切实保护好野生动植物资源。在农林交错地区推广乌伊岭、红星林业局吸收农民参与管护经营的做法。加强森林防火工作，强化重点火险区的治理，提高病虫鼠害科技防治水平，确保森林资源安全。

切实搞好森林培育。适应市场需求，增加苗木品种，加强苗圃行业管理。调整更新方式，增加人工促进更新和人天混更新比重。优化林种树种结构，增加红松、水曲柳、紫椴、黄菠萝、色木槭等珍贵树种的造林比重，大面积营造混交林、阔叶林、速生丰产林、原料林和经济林，完成森工和地方更新造林17万亩，红松和混交林比例达到25%以上。严格执行森林抚育技术规程，提高森林质量和林地生产力，完成森林抚育89万亩。抓好金山屯、乌伊岭、铁力局森林经营综合改革试点工作，为参与国有林权制度改革试点的职工经营林地提供指导和服务。

加强自然保护区和湿地建设。做好生态功能区区划和现有自然保护区晋档升级工作。优化整合自然保护区体系结构，加强丰林、凉水国家级自然保护区建设，努力改变友好湿地、红星湿地、乌伊岭湿地、翠北湿地等省级自然保护区的孤岛化状态，力争建成整体连片、功能齐全的大型国家湿地保护区。

（二）进一步深化改革开放，不断增强发展活力

扎实推进国有林权制度改革试点工作。认真抓好承包后的营造、管护和后续经营工作。及时完成承包经营林地5万亩造林任务，做好承包户地块界限标志设置工作，编制森林经营方案，发展林下经济，实现以短养长。抓好改革配套政策和措施的落实，争取国家尽快出台《林权制度改革试点森林资源管理办法》、《林权制度改革试点林木采伐管理办法》，解决林权证、林木保险、林地经营贷款等难题，解除职工后顾之忧。适时启动活立木交易市场，促进林木资产的有序流转。加强服务体系建设，引导承包职工组建股份林场、合作林场、家庭林场、农林合作社、行业协会等新型经济合作组织，提高森林集约化经营水平。建立森林评估中心、森林托管经营中心、科技服务中心、法律服务中心等社会中介组织，全方位为职工群众提供服务。做好迎接国家林业局专家组对国有林权制度改革试点的评估验收准备工作，为争取进一步扩大试点范围奠定良好基础。

继续推进国有企业改革。深化国有中小企业产权制度改革，对没有完成改制的企业，加快改组、出售和实行股份制。做好西钢、制药企业改制的后续工作，侧重在提高企业经济运行质量和效益上下功夫。做好晨明水泥厂破产清算工作。抓住国家出台木材加工等企业关闭破产的政策机遇，争取将森工院墙、林机修理修配、物资供应和木材经销运输等企业纳入破产范围。

全面推进事业单位改革。对现有事业单位进行科学分类，整合资源，转换用人机制，全面推行聘用和公开招考制度。深化事业单位分配制度改革，建立体现岗位绩效的分配办法和分类分级的调控体制。推行事业单位养老、医疗、失业等社会保险制度。推进财政对事业单位投入机制的改革，年底前全面完成事业单位改革任务。

进一步扩大对外开放。继续实施开放带动战略，以“绿色伊春”为品牌，大力发展开放型经济，改进招商方式，搞好专题性和专业性招商，组织参加好“哈洽会”、伊春（深圳）招商推介会等重大招商引资活动，抓好签约招商项目的落实。争取承办“2007年全球森林生态保护论坛”，办好伊春建市50周年庆典活动，进一步提升伊春的知名度。积极拓展外贸合作领域和空间，扩大出口产品的种类和规模，促进对外贸易协调稳步增长。抓住我国在俄举办“中国年”的契机，全面提升对俄森林采伐、木材加工、农业生产、劳务输出、跨国旅游、建筑市场等领域的合作水平。

（三）调整优化经济结构，强力推进林区经济转型

实施项目牵动战略，做强做大工业经济。谋划和建设一批关系全市工业经济增长后劲的新项目、大项目。整合提高木材精深加工业，突出发展冶金建材化工及矿产开发、绿色能源等特色工业。完成工业项目投资14亿元，投资千万元以上项目12个，开发技术创新项目22个。继续实行重大项目推进机制，扶强晟宇、友春、龙乡、侨艺等家具、人造板和木制工艺品企业，大力开发绿色环保产品，提高市场占有率。支持西钢120吨转炉等项目改造，开工建设浩水二期工程、翠宏山铁矿、桃山小城山风力发电、绿桦水电站等一批重点项目。抓好朗乡耳朵眼山风力发电、南岔庆丰、石头河水电站和兴安太阳能光伏电池等一批续建项目，使其尽快竣工投产。对带岭石顶峰风力发电、兴安红高档红酒、浩化甲醇等一批投产项目，跟踪问效，确保达产达效。继续搞好工业区建设，进一步完善道路管网、给排水、供热、供电、通讯等基础设施，服务入驻企业加快发展。

突出生态旅游，大力发展现代服务业。以“中国森林之都--伊春”为整体形象，以森林避暑度假旅游为主打产品，整合资源，实施旅游精品战略。加强旅游资源规划、保护和管理，加快风景道开发建设，抓好林海奇石风景区、恐龙地质公园、金山鹿苑和溪水森林公园等重点旅游景区建设，完善美溪回龙湾、乌马河梅花河等重点度假区的基础服务设施。提升夏季避暑度假旅游产品档次，深度开发冬季冰雪、野生动物饲养观赏和狩猎旅游等产品，努力在产品创新和宣传促销上实现新突破。继续办好森林生态旅游节和五花山观赏节、冬季摄影节，扩大伊春森林生态旅游知名度。加强旅行社和导游员队伍建设，规范旅游市场秩序。发挥旅游业的带动作用，积极发展现代服务业，引进和培育物流龙头企业，改造提高物流、商贸连锁、餐饮等传统服务业。加快发展金融、保险、中介、会展等现代服务业，大力培育物业管理、家政服务等社区服务业，努力提高第三产业占生产总值的比重。

加大扶持服务力度，壮大非国有经济。落实鼓励非国有经济加快发展的政策措施，加快中小企业自主创新步伐，培植民营企业大户，扩大非国有企业规模，重点帮助指导300户民营规模企业解决好低速运行问题。对全市民营企业计划新上和技改的85个重点项目，给予重点支持，跟踪服务，尽快建设投产，力争5-6户企业的产值超亿元，增强对全市经济拉动作用。抓住我市被列为国家级创业服务试点城市的契机，进一步推动创业富民工程扎实有效开展。采取有效措施，启动内资开发项目。大力发展职工自营经济，使职工自营经济上层次上水平。

（四）转变经济增长方式，提高经济运行质量和效益

增强科技创新能力，加快经济增长。加大科技为生态建设、林权制度改革和经济转型等重点工作的服务力度。抓好朗乡营林工程技术研究中心和带岭森林资源可持续经营技术中试基地建设。支持企业与高等院校、科研院所共建一批产学研紧密合作的产业技术研发服务平台，重点攻关制造业信息化、创新药物和中药现代化、绿色食品和农畜产品精深加工技术等科技难题。调动科技人才的积极性和创造性，完成市级以上重点科技项目30项。大力发展民营科技企业和高新技术企业，引进推广先进实用技术，改造升级传统产业，创新发展接续替代产业。

推进节能降耗，积极发展循环经济。强化资源节约意识，建设节约型企业、社区、学校和机关。大力调整能源消费结构，开发和推广能源节约、替代和循环利用技术，修订行业节能、节水、节材、节地标准，组织实施余热利用、热电联产等一批节能工程。引导林区职工改变生活习惯，增加煤、电、气、太阳能等非木燃料使用的比重，逐步减少对林木资源的消耗，结合林场所住房建设，推广新型节能取暖方式。充分利用采伐、造材、加工剩余物和次小薪材，提高木材综合利用率。以林木资源循环开发利用试点为契机，谋划和建设一批循环经济重点项目，推进以新青为中心的北部木材综合利用循环经济区和以西钢废弃物利用为核心的西林循环经济区建设，形成低投入、低消耗、低污染、高效益的增长方式，努力实现速度、质量、效益、资源、环境的协调统一。

（五）统筹城乡协调发展，加快建设社会主义新农村

加快发展现代农业。坚持用先进的物质条件装备农业，用先进的科学技术改造农业，用先进的经营方式发展农业，用先进的发展理念指导农业，提高农业水利化、机械化、信息化、产业化、标准化和规模化水平，进一步增强农业综合生产能力。大力发展高效农业、品牌农业、生态农业和森工多种经营，积极发展农村二、三产业。搞好山野菜、山野果和菌类特色加工，增加产品的绿色和有机食品认证数量。大力发展生态畜牧业，稳步发展常规养殖，扩大特种养殖，支持翔宇集团、林海肉食制品、盛中乳品、鹿恩堂等龙头企业发展壮大。加强北药资源保护和培育，提升地道优势药材生产能力，打造红星平贝、嘉荫水飞蓟之乡，建立双丰、铁力和桃山五味子种植示范基地，扶持红叶、格润和伊春药业等龙头企业发展，壮大特色种养与加工业。发展各类新型农村合作组织，加快农村经济发展。

加强农村基础设施建设。加大农业投入力度，搞好农田水利基本建设。推广沼气利用，逐步解决农村能源供应问题。继续实施乡镇集中供水、供电、通讯和通畅通达工程，解决农村饮用水、用电、行路难等问题，努力改善农村生产生活条件。

大力发展农村公共事业。加强对农民的宣传教育和技能培训，培育有文化、懂技术、会经营的新型农民，提高农民综合素质，促进农民灵活就业，积极发展打工经济。扩大农村最低生活保障范围，完善新型农村合作医疗制度，改善乡镇卫生院、村屯卫生所医疗设备，提高医疗水平。推进村务公开，强化民主管理。

（六）加强财税金融工作，千方百计增加财政收入

切实做好财政工作。强力推进财源建设，把招商引资、发展接续产业同财源建设紧密结合起来，发挥财税政策和财政资金的导向作用，支持发展兴市、兴区的财源项目，增强财政实力。调整优化支出结构，从严从紧控制一般性支出，重点保障教育、科技、农业等项支出法定增幅，提高社保、下岗再就业支出比重，合理安排公益性基础设施建设和环境保护等支出。严格控制机关事业单位人员编制，对超编进人等违规行为严肃处理。加强非税收入管理，落实收支两条线规定，认真执行国库集中支付和政府采购制度。严肃财经纪律，强化地方财政和森工企业财务管理的审计监督和绩效评价。

加强税收征管。严格执行税收法律法规和政策，完善税收管理制度，做到依法征税，应收尽收，足额入库，防止税收流失。加快税收征管信息化建设，强化重点税源监控，挖掘潜力，增加税收。发挥税务稽查作用，堵塞漏洞，严厉打击偷逃骗税行为，维护税收征管秩序。

优化金融生态环境。抓住国家出台豁免东北老工业基地企业历史欠税的政策机遇，做好免税的争取工作。引导金融机构创新服务方式，提高服务质量，加大对地方经济的支持力度，解决信贷需求难题。进一步探索建立中小企业信用担保体系，提升企业融资能力。引导企业通过市场资本运营，逐步提高直接融资比重。全面创建金融安全区，严厉打击各种逃废金融债务的不法行为。

（七）加强建设与管理，增强城市聚集和辐射功能

完善城市基础设施。继续加大中心城区改造建设力度，深入实施“四区连片”规划，完善新区基础设施。改造升级老城区部分道路、景观、供热管网等市政设施，抓好夜景照明工程建设。进一步完善城市路网结构，完成黑嘉公路嘉荫段改扩建、嘉荫县保兴山支线续建工程和鹤岗至哈尔滨高速公路伊春段、绥嘉公路嘉荫至保兴段、鹤嫩公路伊春至绥棱段项目的前期工作，扩建市客运总站，建成伊春区东、西、北出口客运分站。铺设兴安湖水库城市供水管道，加快中心城污水处理厂、垃圾处理厂建设。完成国家级山洪防御试点项目，推进城市防洪工程建设。做好伊春林都机场“三通一平”等各项准备工作，争取上半年开工建设。

积极推进小城镇建设。依据各地的特点和产业发展状况，做好小城镇总体规划和土地利用规划，加大小城镇改造建设力度，进一步完善交通网、信息网和住宅、排水、环卫等基础设施。积极争取国家政策，加快棚户区和危房改造步伐。大力发展城镇经济，培育壮大主导产业，拉动城镇规模不断扩大。

进一步提高城市经营管理水平。引入市场机制，实施资产运营，把经营性土地、基础设施、公共服务设施以及冠名权、广告权等资产逐步推向市场，拓宽城市建设融资渠道。加强城市管理，进一步完善市与区合理分工、规范高效的城市管理框架。以完善城市基层管理和服务网络为着力点，全面推进和谐社区建设。实行建设项目环保第一审批权，加大城市环境综合整治力度，切实抓好污染减排，进一步改善城市环境。

（八）高度关注民生，全面推进和谐社会建设

进一步完善就业和社会保障体系。采取劳动者自主择业、市场调节就业和政策促进就业等措施，广开就业渠道，统筹做好城镇新增劳动力就业、下岗失业人员再就业、零就业家庭就业工作。做好退役军人就业指导和服务工作。加强劳动力的职业技能培训、创业培训和再就业培训，推行“三年劳动合同计划”，巩固完善和谐劳动关系，提高就业再就业的稳定率，确保新增就业岗位3.5万个。推进城镇单位和企业职工养老、医疗、失业、工伤、生育五大社会保险的扩面工作，加强劳动保障社区平台和信息化建设，严格社保资金管理。完善低保制度，提高城镇居民低保标准。建立健全工资协商和调解机制，促使民营企业劳动者不断提高工资水平。开展好第四轮扶贫解困献爱心活动和城镇困难群体就医、贫困家庭子女入学资助等社会救助工作。医疗、失业、工伤、生育的“四险”补助分批到位，森工企业混岗人员和混岗知青一次性安置的费用足额发放到位，13个政企合一区（局）机关事业单位在职及离退休人员调资工作力争上半年完成。同时，市政府将对森工企业职工工资进行增调。提高林业职工收入水平，改善人民群众生活条件，作为政府责无旁贷！

大力发展各项社会事业。以全面提高教育教学质量为重点，加大教育投入，改善办学条件。巩固九年义务教育成果，大力发展中高等职业教育，支持发展民办教育和继续教育。编写使用伊春乡土教材，教育培养学生在将来发展中成为情系林区、热爱家乡的有根之人。发展公益性文化事业和文化产业，抓好社区、林场所、乡村文化设施建设，加强对12处市级文化遗址的保护和利用，加大网吧等文化市场监管力度，广泛开展“大森林之声”等系列文化活动，不断满足人民群众日益增长的文化需求。扩大伊春电视网络覆盖面，努力解决林场所收看伊春电视节目难的问题。加速推进信息化建设，办好“城市门户网站”。深入实施《全民健身计划纲要》，开展“百万青少年上冰雪”活动，加强体校和青少年俱乐部建设，办好第八届全市运动会。加强医疗卫生工作，开展食品安全专项整治，突出抓好学校、餐饮业和生活饮用水的卫生监督，保障人民群众健康安全。发挥好各类社会组织在促进和谐社会建设中的作用。做好第二次农业普查和计划生育、老龄、外侨等各项工作。

加强社会主义精神文明建设。以践行社会主义荣辱观为重点，深入贯彻《公民道德建设实施纲要》，切实提高公民的思想道德素质。以深化“四自五爱”工程为重点，完善学校、家庭、社会三结合的教育网络，加强和改进未成年人的思想道德建设。以和谐社区、和谐家庭建设为重点，广泛开展形式多样的群众性精神文明创建活动，不断提高社会文明程度。做好“双拥”工作，争创“国家级双拥模范城”四连冠。

全力维护社会稳定。扎实开展“五五”普法宣传教育，提高公民法制观念。深入开展“平安伊春”创建活动，提升社会治安综合治理水平，严厉打击违法犯罪活动，增强群众的安全感。高度重视信访工作，认真落实《信访条例》。统筹协调各方利益关系，积极预防、妥善处置各类群体性事件。加强城镇防火工作，预防交通、生产等重大事故发生。完善突发公共事件应急管理和处置机制，落实各项应急预案和责任制，保障人民群众生命财产安全。

三、切实加强政府自身建设

全面完成今后五年的工作任务，新一届政府必须以振兴伊春为己任，牢固树立正确的政绩观，全面履行执政为民的根本宗旨，不断加强政府自身建设。

增强学习意识，提高施政水平。适应新形势，面对新任务，大力提倡全员学习、终身学习，努力打造学习型政府。全面学习和准确掌握政策法规、经济理论、现代科技、金融外贸等方面的知识，更加注重向实践学习、向基层学习、向群众学习，特别是要增强对新知识、新技术的敏感性，善于捕捉和把握经济发展的先机。创新方式，务求实效，做到学以立德、学以增智、学以致用。自觉运用理论和实践知识指导政府工作，不断增强统揽全局、科学决策、驾驭市场经济的能力。

转变政府职能，创优发展环境。努力改进政府管理经济的方式，完善市场监管机制，依法管理和规范社会事务，提高公共服务水平。完善行政服务中心基础设施和功能，有效发挥行政服务中心的作用，坚决把所有审批事项及收费纳入中心办理，实行“一个中心对外、一个窗口受理、一条龙服务、一站式办结”，为办理各种审批手续提供快捷便利的服务。继续加大纠风力度，对影响经济发展环境的行为，一经发现，严惩不贷。

自觉接受监督，推进依法行政。大力推行政务公开，不断健全重大事项社会公示和听证制度，提高政府工作的透明度。自觉接受人大的法律监督和政协的民主监督，认真办理人大代表议案、建议和政协委员的提案。深入贯彻《行政许可法》，按法定权限和程序办事，依法履行职责。严格落实行政执法责任制、过错追究制，坚决纠正有法不依、执法不严、监管不力等行为。加强行政执法监督和行政复议工作，维护行政执法相对人的合法权益，做到严格执法、公正执法、文明执法，努力提高政府工作的执行力和公信度。

改进工作作风，狠抓工作落实。各级政府必须把工作的着力点放在真抓实干上，鼓实劲不务虚功，求实效不图虚名，开拓进取不自满，埋头苦干不张扬，在狠抓落实中更好地为发展服务，为基层服务，为群众服务。坚持和完善考核、督查通报和责任追究制，健全抓落实的工作机制，确保市政府各项决策部署不折不扣地落到实处。

坚持从严治政，建设廉洁政府。深入实施《公务员法》，加强公务员管理。加大对政府各级干部，特别是领导干部的廉洁自律教育力度。强化行政监察，严厉查处行政违法违纪案件，着重解决森林资源管理、木材生产和销售等领域中的腐败问题。认真落实党风廉政建设责任制，严格遵守廉政建设的各项规定，规范从政行为，树立人民政府为民、务实、清廉的形象。

各位代表、同志们，全力推进伊春经济社会又好又快发展，是时代的召唤、人民的期望、历史的重托。面对未来五年的发展蓝图，我们既感到任务艰巨，责任重大，更感到前景美好、信心百倍。让我们在市委的领导下，以饱满的热情、昂扬的斗志、创新的精神、扎实的工作，攻坚克难，励精图治，为全面建设生态良好、产业发达、体制先进、生活富裕、环境优美、社会文明的新林区而努力奋斗！
